# The Mechanism of Fluorescence Quenching of Protein Photosensitizers Based on miniSOG During Internalization of the HER2 Receptor

**Published:** 2018

**Authors:** E. O. Kuzichkina, O. N. Shilova, S. M. Deyev

**Affiliations:** Shemyakin–Ovchinnikov Institute of Bioorganic Chemistry, Russian Academy of Sciences, Moscow, 117997 , Russia

**Keywords:** targeted protein photosensitizers, internalization, miniSOG, HER2 receptor, fluorescence

## Abstract

The protein photosensitizer miniSOG is a promising agent for photodynamic therapy. The genetically encoded phototoxins 4D5scFv-miniSOG and DARPin-miniSOG specifically bind to the HER2 receptor overexpressed on the surface of cancer cells and promote receptor-mediated internalization of HER2. We show that ingestion of proteins in a complex with the receptor reduces the fluorescent signal of the phototoxic module in endosomes. In order to clarify the mechanism of decrease in the fluorescence intensity of miniSOG-based proteins as they enter a cancer cell during internalization, we analyzed the influence of different factors, including low pH, proteolysis, cofactor reduction, and shielding, on changes in the fluorescence of photosensitizers. Shielding and absorption of miniSOG fluorescence by cell fluorophores, including cytochrome c, were found to contribute significantly to the changes in the fluorescent properties of miniSOG.

## INTRODUCTION

The importance of a targeted delivery of anticancer agents in photodynamic therapy in modern theranostics is on the increase. This approach allows one to enhance the selective accumulation of a photosensitizer in the tumor and deliver it to the desired intracellular compartment [1, 2]. Monoclonal antibodies, antibody fragments, and other proteins capable of selective binding to tumor antigens can be used as targeting fragments.

The cell surface receptor HER2/neu, also known as ErbB2, is an important tumor marker and the best studied target for designing novel therapeutic agents, since it is overexpressed in many tumor types (including human breast cancer cells) and is associated with the aggressive tumor phenotype [3, 4].

The genetically encoded targeted phototoxins 4D5scFv-miniSOG [5] and DARPin-miniSOG [6] were designed and characterized at the Laboratory of Molecular Immunology of the Institute of Bioorganic Chemistry, Russian Academy of Sciences. A fragment of monoclonal antibody 4D5scFv and the artificial protein DARPin_9-29, capable of selective recognition of the extracellular domain of human epidermal growth factor receptor HER2/neu, were employed as targeting modules. In both cases, photoactivatable fluorescent flavoprotein miniSOG was used as a phototoxic module [7]. Both proteins exhibited a selective phototoxic effect in in vitro experiments: in HER2-positive human breast adenocarcinoma SK-BR-3 cells, IC_50_ was equal to 160 and 0.8 nM for 4D5scFv-miniSOG and DARPin-miniSOG, respectively. Furthermore, both of these proteins were capable of inducing receptor-mediated endocytosis [4-6]. However, the internalization rate of the DARPin-miniSOG–HER2 complex was higher than that of the 4D5scFv-miniSOG–HER2 complex [8]. The dissociation constants of the phototoxins and the receptor measured by surface plasmon resonance are comparable. Therefore, a conclusion has been drawn that the internalization rate, which determines the residence time of the toxin on the membrane, makes the most significant contribution to the efficiency of these photosensitizers.

It is possible to rapidly assess the dynamics of internalization of these proteins due to the fact that miniSOG exhibits intrinsic fluorescence, with its intensity decreasing as phototoxins enter the endosomes. However, the mechanism of fluorescence quenching in miniSOG upon entering the cell has not been elucidated yet. Based on the processes taking place in the endosome, several hypotheses can be formulated to interpret this phenomenon. This fluorescence quenching of some fluorophores in the endosome can be related to protonation, as pH is decreased. For example, fluorescein isothiocyanate (FITC) responds to changes in acidity and is used to study the internalization of cell receptors [9]. The miniSOG chromophore is based on a flavin mononucleotide (FMN) that can also be protonated; so, this can be the reason for the fluorescence decline [9, 10]. The fluorescent properties of the miniSOG protein depend on its cofactor; therefore, it is less likely that proteolysis taking place in the endosome or lysosome is the cause of this phenomenon. Finally, the fluorescence intensity of phototoxins can be reduced as chromophores in the cytoplasm shield miniSOG and absorb its fluorescence.

This study focused on the causes of the reduction in the fluorescence intensity of the phototoxic proteins 4D5scFv-miniSOG and DARPin-miniSOG. Fluorescence quenching of miniSOG in the endosome makes this module a convenient tool for investigating internalization dynamics. However, it is important to understand the reasons for this phenomenon, since the fluorescent properties of miniSOG are closely related to its toxic properties. Furthermore, when designing systems for phototoxin delivery to therapeutic targets, researchers should take into account the physicochemical processes with the participation of miniSOG that take place in different cellular compartments.

## MATERIALS AND METHODS

**Cell lines and culture conditions**

Chinese hamster ovary (CHO) cells and human breast adenocarcinoma SK-BR-3 cells overexpressing the cell surface receptor HER2 were cultured in the McCoy’s 5A medium (Life Technologies, USA) supplemented with 10% fetal bovine serum (FBS) (HyClone, Belgium) and 2 mM *L*-glutamine (PanEco, Russia) in atmosphere of 5% CO_2_ at 37°C.

**Production of recombinant proteins 4D5scFv-miniSOG and DARPin-miniSOG**

Proteins 4D5scFv-miniSOG and DARPin-miniSOG were produced in *Escherichia coli *strain BL21(DE3). The cells were transformed using a pET22b plasmid carrying the gene of the respective protein. The transformed bacteria were cultured in a LB liquid medium (1% tryptone, 0.5% yeast extract, and 1% NaCl) until the optical density OD_600_ reached 0.5. Expression was induced by 1 mM isopropyl β-*D*-1-thiogalactopyranoside (IPTG, Merck, Germany); the biomass was then grown at 25°C for 24 h. The resulting biomass was precipitated by centrifugation (10,000*g*) at 4°C for 10 min, re-suspended in 60 mL of 20 mM phosphate-buffered saline (3.2 mM NaH_2_PO_4_, 16.8 mM Na_2_HPO_4_, 0.3 M NaCl, pH 7.5), and subjected to ultrasonic lysis using a VCX120 sonicator (Sonic and Materials Inc., USA) in the pulsed mode (pulse for 30 s, cooling down for 30 s; 70% amplitude) for 5 min. In order to separate the soluble and insoluble fractions, the lysate was centrifuged (50,000*g*) at 10°C for 30 min. The precipitate was separated from the supernatant liquid, and the target proteins were isolated.

Proteins 4D5scFv-miniSOG and DARPin-miniSOG were isolated from the soluble fraction by metal-chelate affinity chromatography on a HisTrap FF 1 mL column (GE Healthcare, USA) loaded with Ni^2+^-NTA-sepharose. The proteins were eluted via stepwise increase in imidazole concentration from 15 to 500 mM in 20 mM phosphate-buffered saline (pH 7.5) at an elution rate of 0.5 mL/min using a UV cell (RD2:250-280, Reach Devices, USA) detecting light absorption at 260 and 280 nm. After chromatography, the protein fractions were analyzed by sodium dodecyl sulfate polyacrylamide gel electrophoresis (SDS-PAGE) under denaturing conditions according to the Laemmli’s protocol. Concentrations of the target proteins were determined by the biuret test in the presence of bicinchoninic acid using a Pierce BSA Protein Assay Kit (Thermo Scientific, USA), in compliance with the manufacturer’s protocol.

**Verification of the specificity of 4D5scFv-miniSOG and DARPin-miniSOG binding to the HER2 receptor**

The presence of the HER2 receptor on cells and specificity of binding of the 4D5scFv-miniSOG and DARPin-miniSOG proteins to HER2 were analyzed using a BD Accuri C6 flow cytometer (Becton Dickinson, USA) with the following parameters: laser power, 20 mW; wavelength, 488 nm; and filters 533/30 BP (the FL1 channel) for detecting protein fluorescence and 585/40 BP (the FL3 channel) for detecting fluorescence of propidium iodide. The data were analyzed using the BD Accuri C6 software and processed using the FlowJo program. HER2-positive SK-BR-3 cells and HER2-negative CHO cells were used in the experiment.

Samples consisting of ~10^5^ cells were incubated with the proteins 4D5scFv-miniSOG, DARPin-miniSOG, and 4D5scFv conjugated to FITC (4D5scFv-FITC) or with FITC-conjugated beta-lactoglobulin (β-LG-FITC) (all proteins were taken at concentration of 2 μm) in PBS supplemented with 1% bovine serum albumin (Dia-M, Russia) on ice during 15 min. After staining, the cell suspension was washed twice with PBS supplemented with 1% bovine serum albumin. To eliminate non-living cells from the analysis, the sample was incubated with 2.5 μg/mL propidium iodide for 5 min prior to measurements. When performing fluorescence measurements, single-cell populations were isolated according to light scattering parameters (FSC-H/FSC-A). Among them, live cells not stained with propidium iodide were selected for the analysis. At least 10^4^ fluorescent events were recorded for each sample.

**Studying the internalization rate of DARPin-miniSOG and 4D5scFv-miniSOG**

SK-BR-3 cells stained with DARPin-miniSOG and 4D5scFv-miniSOG were used to evaluate the rate of receptor-mediated internalization. The fluorescence of the samples was measured on a BD Accuri C6 flow cytometer (Becton Dickinson, USA) according to the procedure described above. The samples were subdivided into two groups. In the first one, the samples were incubated at 4°C after staining (receptor-mediated internalization does not occur under these conditions). The samples in the second group were incubated at 37°C. The readings were taken at several time points: 5, 10, 30, and 60 min. At least 10^4^ events were recorded for each sample.

**Studying the mechanism of quenching of miniSOG-based phototoxins in the endosome**

The effects of pH and proteases on DARPin-miniSOG, 4D5scFv-miniSOG, and FMN were evaluated by measuring the intensity of the fluorescence induced by light with λ = 488 nm and detected at λ = 535 nm on an Infinite M1000 microplate reader (Tecan, Switzerland). The proteins at a concentration of 35 μm were incubated at 37°C in a buffer (100 mM Tris-HCl), with pH brought to the desired value, containing proteolytic enzymes at a concentration of 40 μM (trypsin, chymotrypsin, pepsin, and papain) (Sigma, USA) and reducing agents (dithiothreitol, glutathione (reduced form), ascorbic acid, NADH, NaBH4 (Sigma, USA)) at concentrations of 10 mM. The readings were taken immediately after adding the protein and after incubation for 1 and 2 h.

A Trypan blue dye (PanEco, Russia) at a 0.1% concentration and cytochrome *c *at a 300 μM concentration (Sigma, USA) were used to evaluate the effect of the presence of other chromophores on the fluorescence intensity of FMN and DARPin-miniSOG as compared to that of DARPin-FITC. The fluorescence intensities of the samples were measured on an Infinite M1000 microplate reader (Tecan, Switzerland). Fluorescence was excited by light (λ = 488 nm) and detected in a wavelength range of 525–545 nm. The measurements were made immediately after the chromophores had been added.

## RESULTS AND DISCUSSION

In order to ensure efficient performance by an antitumor agent in photodynamic therapy, it needs to be selectively delivered to the target. Thus, we used two target molecules specific to the HER2 surface receptor, a non-immunoglobulin protein (DARPin_9-29), and a single-chain variable fragment (scFv) of the 4D5 antibody, to deliver the cytotoxic module miniSOG. The phototoxic module miniSOG is a flavoprotein that can generate reactive oxygen species when exposed to blue light due to the bound FMN. In order to produce recombinant proteins, *E. coli *BL21(DE3) cells were transformed with the respective plasmids pET22b-4D5scFv-miniSOG and pDARPin-miniSOG. The proteins 4D5scFv-miniSOG and DARPin-miniSOG were isolated from the soluble fraction by Ni^2+^-NTA metal-chelate affinity chromatography involving imidazole elution. In order to verify the activity of the resulting protein photosensitizers, the specificity of binding of the 4D5scFv-miniSOG and DARPin-miniSOG recombinant proteins to the HER2/neu receptor on the surface of human breast adenocarcinoma SK-BR-3 cells overexpressing HER2/neu was measured by flow cytofluorimetry. This procedure allowed for testing the selectivity of binding between the targeting module of DARPin and the receptor, as well as flavoprotein functionality, since the toxic module miniSOG exhibits intrinsic fluorescence and its binding to the cells can be detected directly [11].

The presence of the HER2/neu receptor on the cell surface was confirmed by the fact that the cells were stained with fluorescein isothiocyanate-labeled 4D5scFv (4D5scFv-FITC). FITC-labeled β-lactoglobulin (β-LG-FITC), which does not bind to HER2 on the cell surface, was used as a negative control. It was demonstrated that HER2-negative CHO cells do not generate a fluorescent signal after incubation with the protein 4D5scFv-FITC or β-LG-FITC and the target proteins 4D5scFv-miniSOG and DARPin-miniSOG (*Table 1*).

**Table:** Fluorescence of cells after staining with various proteins. The mean values for the three experiments ± mean error are given

Sample	Fluorescence intensity measured in the FL1 channel	
	SK-BR-3 cells	CHO cells
Unstained cells	3700 ± 400	3700 ± 900
+ β-LG-FITC	5700 ± 600	3300 ± 400
+ 4D5scFv-FITC	2.7×10^4^ ± 7 × 10^3^	3200 ± 500
+ 4D5scFv-miniSOG	2.3×10^4^ ± 3 × 10^3^	4600 ± 400
+ DARPin-miniSOG	1.71×10^4^ ± 1.6 × 10^3^	3000 ± 400

Hence, it has been demonstrated that the targeted recombinant proteins 4D5scFv-miniSOG and DARPin-miniSOG are capable of highly specific binding to the HER2/neu receptor on the surface of human breast adenocarcinoma SK-BR-3 cells.

**Fig. 1:**
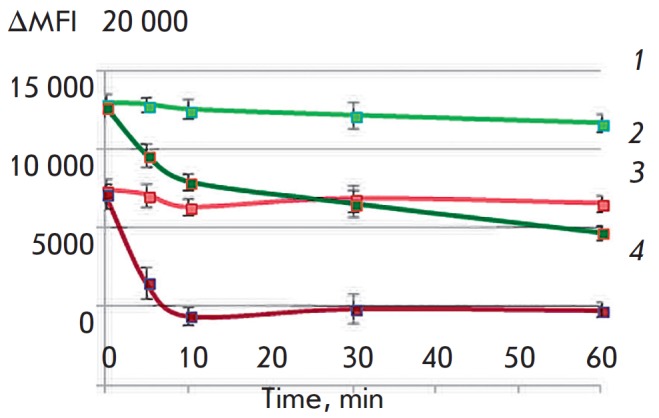
Changes in the fluorescence intensity of phototoxic proteins during internalization in combination with HER2 (+37°C) and under conditions preventing internalization (+4°C). *1 *– 4D5scFv-miniSOG, +4°C; *2 *– DARPin-miniSOG, +4°C; *3 *– 4D5scFv-miniSOG, +37°C; *4 *– DARPin-miniSOG, +37°C. ΔMFI is the difference in fluorescence intensities between the stained cells and the unstained sample incubated under the same conditions

It was revealed that receptor-mediated internalization of proteins did not take place after the DARPin-miniSOG and 4D5scFv-miniSOG proteins were bound to the receptor on the surface of SK-BR-3 cells at +4°C. However, the receptor–protein complex undergoes internalization at +37°C, as evidenced by the reduction in the fluorescence intensity ΔMFI (the difference between the average fluorescence intensities of stained and unstained cells) (*Fig. 1*). The DARPin-miniSOG recombinant protein as part of its complex with the receptor is internalized faster than 4D5scFv-miniSOG, since ΔMFI for DARPin-miniSOG decreases twofold as compared to its baseline during the first 10 min, while ΔMFI for 4D5scFv-miniSOG is 40 min. These findings are consistent with the published data: 4D5scFv-miniSOG has a higher cytotoxicity than DARPin-miniSOG [5, 6], because 4D5scFv-miniSOG resides on the membrane for a longer time. Since necrosis is the predominant death mechanism of cells irradiated in the presence of these phototoxins, membrane damage makes a crucial contribution to the toxicity of targeted proteins. However, the decline in the fluorescence intensity of miniSOG can be indicative of reactions involving chromophore, which is also expected to affect its efficiency as a phototoxin.

**Fig. 2:**
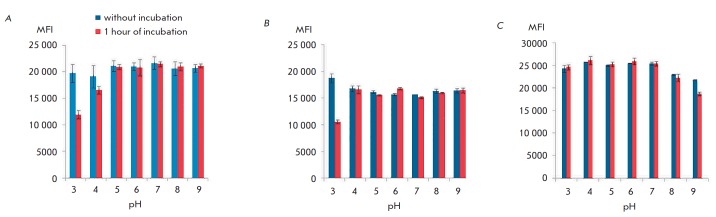
Dependence of the intensity of the fluorescent signals of 4D5scFv-miniSOG (*A*) DARPin–miniSOG (*B*) and FMN (*C*) on the pH of the medium at 37°C for 1 h. The fluorescence intensity was recorded in a wavelength range of 525–545 nm on an M1000 Pro microplate reader (Tecan, Switzerland). Here and in *Fig. 3*, *Fig. 4*, *Fig. 5*, MFI is the fluorescence intensity; M ± m, *n *= 3

In order to elucidate the reasons for the decline in the fluorescence intensity and toxicity of miniSOG-based proteins observed during their internalization, we evaluated the effect of various factors on the fluorescent properties of miniSOG. A hypothesis has been put forward that quenching of DARPin-miniSOG fluorescence during internalization can be associated with changes in the pH of the environment, as the receptor–protein complex enters endosomes and lysosomes. *Figure 2* shows the dependence between the fluorescence intensity of the DARPin-miniSOG and 4D5scFv-miniSOG proteins and the flavin cofactor (FMN) on the pH of the solution at +37°C. A reliable and significant decline in fluorescent intensity (over twofold) was observed at pH 3 and a less pronounced decline was detected at pH 4. Meanwhile, the minimal pH in the endosomes and lysosomes is 4.8 [12]. Therefore, quenching of miniSOG fluorescence during its internalization cannot be attributed to its response to endosomal acidification. Furthermore, variation in the temperature in a range from +4° to +37°C is not the reason for the decline in the fluorescence intensity of FMN and DARPin-miniSOG.

**Fig. 3:**
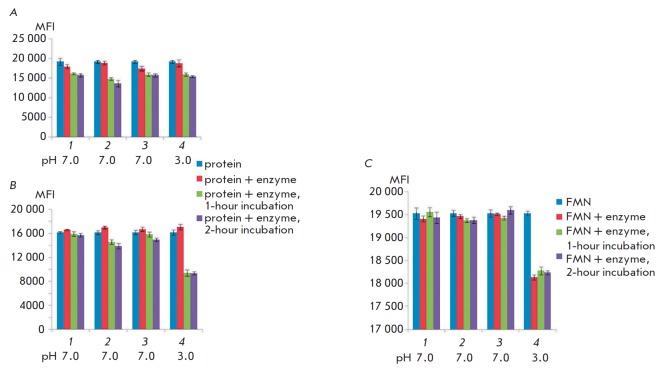
Dependence of the fluorescence intensity of 4D5scFv-miniSOG (*A*), DARPin–miniSOG, (*B*) and FMN (*C*) on treatment with specific proteases at 37°C for 1 h. *1 *– trypsin; *2 *– chymotrypsin; *3 *– papain; and *4 *– pepsin. The fluorescence was recorded in a wavelength range of 525–545 nm

In order to test the hypothesis regarding the possible effect of proteolysis in the endosome on the fluorescence intensity of DARPin-miniSOG and 4D5scFv-miniSOG, we simulated the conditions of proteolytic cleavage by such enzymes as trypsin, papain, and chymotrypsin that is similar to endosomal cathepsin G [13], and pepsin. The activity of the latter enzyme was comparable to those of the lysosomal cathepsins D and E at pH values optimal for them. FMN was used as a control. A significant decline in the fluorescence intensity of FMN, DARPin-miniSOG, and 4D5scFv-miniSOG was observed only when the target proteins were treated with pepsin (*Fig. 3*). However, the data presented above (*Fig. 2*) provide grounds for inferring that a low pH is the reason for fluorescence quenching. This hypothesis is also supported by the fact that the fluorescence intensity decreased in the reaction mixture that contained FMN (insensitive to proteolysis), instead of phototoxic proteins. Treatment with other proteases did not reduce the fluorescence intensity twofold or more, while such a reduction was observed upon internalization (*Fig. 1*). Cleavage of DARPin-miniSOG and 4D5scFv-miniSOG under the experimental conditions was confirmed by PAGE (15% PAG), using the Laemmli protocol. Phototoxic proteins were fully cleaved after 1-hour incubation.

**Fig. 4:**
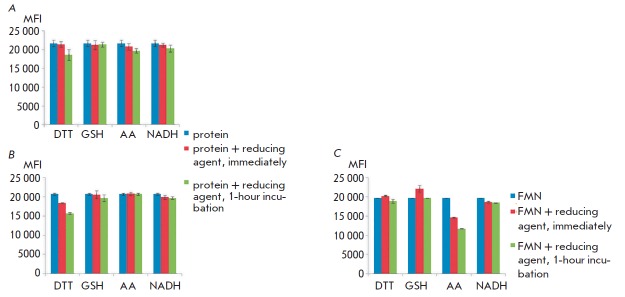
Dependence of the intensity of the fluorescent signal of 4D5scFv-miniSOG (*A*), DARPin–miniSOG (*B*), and FMN (*C*) on the effects of intracellular reducing agents at 37°C for 1 h. DTT – dithiothreitol; AA – ascorbic acid. The fluorescence was recorded in a wavelength range of 525–545 nm

Another reason for the decline in the fluorescence intensity of miniSOG during receptor-mediated internalization could be based on the fact that the flavin mononucleotide cofactor was reduced by the reactive molecules in the cell. Reduction of FMN is known to decrease its intensity [15]. In case a significant percentage of the oxidized form of the cofactor is ingested by the protein during production of miniSOG in bacteria or the cofactor is easily oxidized upon storage, its reduction can be responsible for quenching of the fluorophore in the cell. The effects of the following reducing agents on DARPin-miniSOG and 4D5scFv-miniSOG were studied: dithiothreitol, glutathione (in its reduced form, GSH), ascorbic acid, NADH, sodium borohydride (NaBH4) (*Fig. 4A,B*), and FMN in the absence of the protein component (*Fig. 4C*). It was found that unbound FMN can be reduced by NaBH_4_ and ascorbic acid, which leads to an almost twofold decrease in the fluorescence intensity. Meanwhile, reduction of the flavin mononucleotide cofactor within DARPin-miniSOG occurs only at high NaBH4 concentrations (starting from 10 mM). Since the more physiologically relevant reducing agents did not exhibit this effect, a conclusion was drawn that reduction of the cofactor does not significantly contribute to the changes in the fluorescence intensity of miniSOG in the cell. Furthermore, the effect of intracellular reducing agents on the fluorescence intensity of DARPin-miniSOG and 4D5scFv-miniSOG after protease treatment at a pH optimal for these enzymes has been evaluated. This treatment also did not affect the fluorescent properties of the miniSOG-based proteins.

**Fig. 5:**
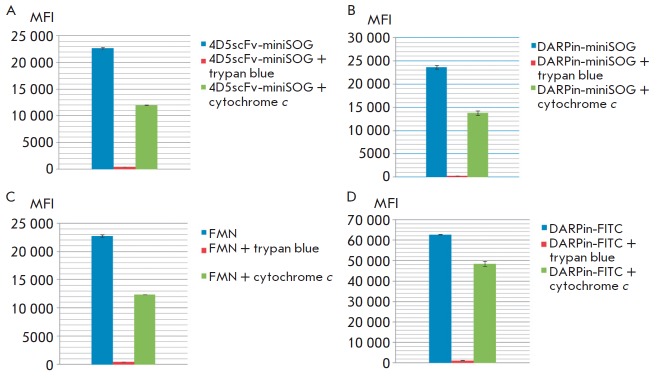
Effect of the presence of different chromophores on the fluorescence intensity of 4D5scFv-miniSOG (*A*), DARPin–miniSOG (*B*) FMN (*C*), and DARPin- FITC (*D*). The fluorescence was recorded in a wavelength range of 525–545 nm

An alternative hypothesis explaining the decline in the fluorescence intensity of miniSOG in the cell is shielding of a protein molecule and quenching of its fluorescence by intrinsic chromophores. The Trypan blue dye was tested as a model molecule capable of *in vitro *quenching of miniSOG fluorescence. The dye contributed to complete fluorescence quenching of both DARPin-miniSOG and 4D5scFv-miniSOG. Hemoproteins, such as cytochrome *c*, can act as native agents that absorb miniSOG radiation inside the cell. It has been demonstrated that when fluorescence is excited in the presence of cytochrome, fluorescence intensity decreases twofold both for FMN and for the target proteins. This effect was not observed for DARPin conjugated to FITC, the conventional fluorescent dye (*Fig. 5*).

It is worth mentioning that FITC fluorescence in endosomes and lysosomes also varies depending on the pH of the environment. This dye is currently used as a sensor for measuring the pH of endosomes in cells [9, 10]. Like FITC, the cytotoxic module miniSOG can be employed to detect HER2 internalization, but the reasons for the decline in fluorescence intensity in these two cases are different.

## CONCLUSIONS

The key reason for the decline in the fluorescence intensity of miniSOG-based phototoxins is their shielding and absorption of miniSOG fluorescence by intrinsic cellular fluorophores. The stability of miniSOG inside the cell makes it a promising component for designing theranostic agents, as its spectral properties make it possible to use it together with NanoLuc luciferase, which solves the problem of miniSOG shielding [16]. We have discovered that the cytotoxic module miniSOG within the recombinant proteins 4D5scFv-miniSOG and DARPin-miniSOG can be used to detect HER2 internalization in the same manner as FITC, but the reasons for quenching of the fluorescent signals are different.

Understanding the mechanism of fluorescence quenching in the photosensitizer allows one to adequately interpret the data on the dynamics of internalization of theranostic agents in a complex with the HER2 receptor. This is of utmost importance for a rational design of targeted phototoxic agents, since their efficiency was earlier found to depend on their localization and accumulation in tumor cells [17].

## Abbreviations

PDT: photodynamic therapy
HER2: human epidermal growth factor receptor 2
scFv: single-chain variable antibody fragment
DARPin: designed ankyrin repeat protein
FITC: fluorescein isothiocyanate
FMN: flavin mononucleotide
IPTG: isopropylthio-?-D-galactopyranoside
PAG: polyacrylamide gel
SDS: sodium dodecyl sulfate
PBS: phosphate-buffered saline
GSH: glutathione
